# Recent Advances in Serum Biomarkers for Cardiological Risk Stratification and Insight into the Cardiac Management of the Patients With Hematological Malignancies Treated With Targeted Therapy

**DOI:** 10.7759/cureus.49696

**Published:** 2023-11-30

**Authors:** Mihaela Andreescu

**Affiliations:** 1 Department of Hematology, Colentina Clinical Hospital, Bucharest, ROU; 2 Department of Clinical Sciences, Hematology, Faculty of Medicine, Titu Maiorescu University of Bucharest, Bucharest, ROU

**Keywords:** risk prediction, screening tools, cancer therapy, cardio protection, cardiotoxicity, serum biomarkers, cardio-oncology

## Abstract

Cardiovascular diseases (CVD) have emerged as a common and serious complication of cancer treatment, particularly in patients undergoing cardiotoxic therapies. Over the last few years, the medical community has become increasingly aware of the potential for cardiotoxicity resulting from cancer treatments involving chemotherapy, targeted therapies, and radiation therapy. This recognition is due to the significant risk of morbidity and mortality in cancer patients and survivors resulting from such treatment-induced cardiovascular damage. While the cardiotoxic effects of chemotherapy and targeted therapy have been discussed in medical literature, only a limited number of studies have explored the role of serum biomarkers in cardiological risk stratification. In recent years, serum biomarkers have emerged as a valuable tool for assessing and managing cardiotoxicity in patients with hematological malignancies. This review article provides a summary of the current state of knowledge on the usefulness of biomarkers in managing cardiotoxicity resulting from different targeted therapies throughout the cancer care continuum. Although cardiac biomarkers have demonstrated potential in identifying subclinical cardiotoxicity and tracking the response to cardioprotective treatments, further research is necessary to determine optimal biomarkers and surveillance strategies. The incorporation of cardiac biomarkers into clinical practice in patients undergoing targeted therapies could potentially lead to improved long-term cardiovascular outcomes in cancer patients and survivors.

## Introduction and background

In recent times, a significant increase in the prevalence of cardiovascular diseases (CVD) has been observed in patients with hematological malignancies (HM) and, in particular lymphoproliferative disorders (LPDs) who are undergoing certain targeted therapies [1]. Although several factors like advanced age, immune system dysregulation, and genetic predisposition have been identified as the potential causes of CVDs in LPDs, recent evidence suggests that certain chemotherapeutic agents, targeted therapies, and radiation treatments may also contribute significantly to CVD-related toxicity [2]. As CVDs have a significant impact on the health outcomes of individuals who have undergone cancer treatment, it is essential to evaluate various risk factors, identify early signs of subclinical disease during and post-cancer therapy, and make prognostic predictions. Furthermore, it is crucial to assess the cardiovascular health of cancer patients before initiating treatment and optimize the management of pre-existing cardiovascular disease, particularly for patients with a documented high risk of CVD. To aid in the initial risk assessment, the Heart Failure Association (HFA) Cardio-Oncology Study Group and the International Cardio-Oncology Society have recently released risk stratification templates for various cardiotoxic therapies, including targeted therapies [3].

Serum biomarkers play a significant role in evaluating the initial risk of CVDs and assist in their early diagnosis [4]. Hence, the elevation of cardiovascular biomarkers such as troponin and natriuretic peptides can aid in the identification of patients who require cardioprotective treatments during cancer treatment. These biomarkers also enable the effective monitoring of the response to cardioprotective treatments and provide valuable prognostic information [5]. An ideal cardiac biomarker should exhibit properties including simplicity of assessment, reliability, and the capacity to precisely anticipate clinical consequences [5]. Furthermore, it should have a substantial impact on guiding the process of risk stratification or the development of treatment protocols. While a considerable body of literature exists on chemotherapy-induced cardiotoxicity, fewer studies have focused on targeted therapies, particularly regarding the use of serum cardiovascular biomarkers for cardiological risk stratification. This review aims to provide a summary of current research on recent advances in serum cardiovascular biomarkers for the assessment of cardiotoxicities in patients with hematological malignancies (HM) undergoing targeted therapies.

## Review

Pathophysiology of serum biomarkers

Biomarkers have potential utility in a range of clinical settings, including disease diagnosis, monitoring, risk stratification, and treatment selection [6]. In the context of CVDs in patients with lymphoproliferative disorders, several serum biomarkers have been suggested for risk stratification, including pro-B-type natriuretic peptide (proBNP), high-sensitivity C-reactive protein (hs-CRP), troponins, and brain natriuretic peptide (BNP). The underlying pathophysiological mechanisms that link these biomarkers to LPDs are intricate and multifactorial.

Cardiac troponin

Troponin is a biomarker that was initially employed for diagnosing acute coronary syndromes but has since been shown to be beneficial in identifying cardiotoxicity. It is a critical constituent of the myofibrillar apparatus in the heart muscle cells, and its existence is distinct from the cardiac myocyte [7,8]. Although some studies suggest that troponin T may also exist in skeletal muscle, troponin I is regarded as the most accurate indicator of cardiac injury. Cardiac troponin T (cTnT) and cardiac troponin I (cTnI) are organ-specific proteins that are unique to the heart. Troponin plays a crucial role in the excitation-contraction process of cardiac muscle cells, and its measurement provides valuable information for assessing cardiac function and damage [9]. The release of cardiac troponins (cTnT, cTnI) varies based on the extent of cardiac insult (necrosis, apoptosis). The process of cardiac troponin release can be described in three steps. First, cTns are released from myocardial cells due to the renewal of myocardial cells. After that, there is a circulation of cTns in the plasma followed by removal from the bloodstream through the glomerular barrier [7]. Therefore, it is crucial to understand that an increase in troponin levels does not always indicate myocardial necrosis and may be a result of various mechanisms. Troponin can be detected in response to myocardial stress, as a consequence of transient rises in cell permeability due to cellular wounds, cytoplasmic blebbing, or apoptosis [10]. Recently, the utilization of hs-cTn has been introduced, enabling the identification of troponin concentrations at remarkably low levels and the detection of circulating levels in as many as 50% of the adult population [11].

Natriuretic peptides

As per the present recommendations from the European Society of Cardiology and the American Heart Association, the biomarkers BNP and NT-proBNP have been accorded a Class IA recommendation for diagnosing heart failure (HF). These natriuretic peptides are the sole biomarkers that have received this recommendation, signifying their high level of clinical utility and importance in the diagnosis of HF [12,13]. In conditions of hemodynamic stress and congestion, there is a notable rise in the levels of BNP [14,15]. The main trigger for the secretion of BNP is myocardial stretching which arises in response to heightened intracardiac volume and pressure. Additionally, transcriptional upregulation of BNP can occur in response to catecholamines and angiotensin-II [16]. Although BNP and NT-proBNP are primarily associated with cardiac myocyte stress, there is some evidence to suggest a positive correlation between NT-proBNP and inflammatory cytokines such as interleukin-6 (IL-6) [17]. Natriuretic peptides (NPs) are also involved in feedback loop mechanisms. As NPs have anti-inflammatory properties, it suggests that inflammation also serves as a stimulus for NP release. Fish-Trotter et al. revealed that a 1-unit higher baseline natural log IL-6 level was associated with a 6% higher NT-proBNP level [17]. The utilization of NPs in screening non-cancer populations with CVD risk factors has shown promise in identifying individuals at greater risk of developing left ventricular dysfunction (LVD), thereby enabling targeted preventive measures. This method could also be employed to detect LVD in high-risk cancer patients undergoing treatments that are associated with the development of LVD and HF [18,19].

Interleukin 6 and high sensitivity CRP 

One of the primary pathophysiological mechanisms leading to an increased risk of CVDs in patients with LPDs is inflammation. Chronic inflammation can promote atherosclerosis, a significant contributor to CVD. CRP is a widely studied serum biomarker for stratifying cardiological risk. It is an acute-phase reactant synthesized by the liver in response to pro-inflammatory cytokines such as IL-6 [20]. Swastini et al. in their study showed that there is a significant association between hs-CRP concentration level and the severity of atherosclerosis (p < 0.01) [21]. Besides enhancing CRP production, IL-6 stimulates endothelial cell proliferation, lymphocyte differentiation, and coagulation, all of which are critical inflammatory mediators contributing to coronary artery disease. HsCRP has been shown to be useful in primary and secondary prevention studies to predict coronary artery disease, plaque instability, myocardial infarction (MI), and stroke [22]. Held et al. have established an independent relationship between IL-6 and MI, all-cause mortality, and heart failure hospitalizations [23].

Interleukin 1 receptor-like 1 (ST2) 

ST2, a member of the Interleukin-1 (IL-1) receptor family, functions as a receptor for the ligand IL-33 in both transmembrane and soluble forms [24]. Initial investigations by Weinberg et al. revealed that ST2 is upregulated in response to myocardial injury and stretching [25]. Unlike transmembrane IL-33, soluble IL-33 impedes the protective effects of IL-33 by sequestering it. Elevated levels of soluble ST2 have demonstrated prognostic significance in predicting mortality in both acute and chronic heart failure. While soluble ST2 may not be a particularly strong indicator of acute myocardial injury, it is associated with the onset of heart failure following non-ST elevation acute coronary syndrome [26]. The use of ST2 can provide prognostic information complementary to that of natriuretic peptides. Therefore, it can be used in conjunction with other cardiac biomarkers [26].

Galectin-3

Galectin-3 (Gal-3) is secreted by monocytes upon their differentiation into macrophages. It plays a crucial role in modulating various inflammatory signaling pathways by interacting with a diverse range of ligands [27]. In rats, it has been observed to induce cardiac hypertrophy through the promotion of fibroblast proliferation and profibrotic effects [28]. However, the relevance of Gal-3 in detecting cardiotoxicity caused by cancer therapy is complicated by its upregulation in various types of cancer [27]. Two meta-analyses have shown that Gal-3 is a promising but contradictory prognostic indicator for mortality from all causes and cardiovascular diseases [29,30]. Srivatsan et al. performed a multivariate analysis which showed Gal-3 is ineffective in predicting all-cause mortality and cardiovascular mortality, especially under the influence of factors such as estimated glomerular filtration rate (eGFR), left ventricular ejection fraction (LVEF) and N-terminal pro-B-type natriuretic peptide (NTproBNP). However, the efficacy of the combination of Gal-3 with other biomarkers was much higher compared to a single biomarker [29]. An analysis of patients with chronic heart failure with preserved, reduced, and recovered ejection fraction found that Gal-3 is significantly associated with severe symptoms of heart failure and adverse events (such as mortality from all causes and implantation of a heart transplant or VAD) [31].

Placental growth factor

Placental growth factor (PlGF) plays a crucial role in the regulation of hypertrophy and angiogenesis processes. PlGF has been identified as a prognostic marker for both all-cause mortality and non-fatal myocardial infarction in patients with acute coronary syndrome and has demonstrated its predictive value in both short-term and long-term follow-up studies [31,32].

Targeted therapies, their cardiotoxicity, and serum cardiac biomarkers

Bruton’s Tyrosine Kinase (BTK) Inhibitors

BTK is a non-receptor member of the TEC kinase family that plays a critical role in oncogenic signaling pathways, including B cell receptor and chemokine signaling [33].

Ibrutinib: Ibrutinib, an irreversible BTK inhibitor, was initially approved by the FDA in 2013 for the treatment of mantle cell lymphoma (MCL) and later in 2014 for chronic lymphocytic leukemia (CLL) and in 2015 for Waldenström’s macroglobulinemia [34]. Multiple studies have demonstrated the potential cardiotoxicity associated with the use of ibrutinib. For example, Ibrutinib increases the risk of atrial fibrillation by inhibiting cardiac phosphoinositide 3-kinases (PI3K)-AKT signaling. Similarly, acute ibrutinib prolongs cardiac action potentials (APs) and triggers abnormal APs [35-37]. The underlying mechanism of ibrutinib-related cardiotoxicity is attributed to PI3K and TEC pathways which are generally involved in cardiac protection. PI3Kα, an isoform of class I PI3K isoforms, plays a significant role in cardiac hypertrophy and contractility and is activated by insulin-like growth factors in cardiomyocytes. Suppression of PI3Kα has been shown to exacerbate hypertrophic cardiomyopathy caused by pressure overload or MI in mouse models, whereas activation of PI3Kα can ameliorate hypertrophic and dilated cardiomyopathy [38,39]. Furthermore, ibrutinib-related inhibition of PI3Kα leads to activation of late Na current (INa-L), which results in QT prolongation [39].

Jiang et al. showed that ibrutinib (25 mg/kg/d) for four weeks resulted in a higher incidence of arterial fibrillation (AF) and reduced Ca^2+^ transient amplitude along with enhanced spontaneous Ca^2+^ release in atrial myocytes compared to a control group [40]. Furthermore, the incidence of AF was measured as 5.77 per 100 person-years when evaluated over a period of 18.3 months follow-up [41]. A review by Neilan et al. showed that the incidence of AF was much higher in subjects who received ibrutinib therapy compared to chemotherapy (6.5% vs. 1.6%, respectively) [42]. Furthermore, the risk of incidence of AF was much higher in CLL patients compared to mantle cell lymphoma (7.0% vs. 4.3%). Several risk factors, such as a history of AF, age over 65 years, and hyperlipidemia, have been shown to have an early onset of ibrutinib-induced AF [42]. Apart from this, prior use of angiotensin-converting enzyme inhibitors, beta-blockers, and aspirin can exacerbate the risk of ibrutinib-induced AF [43]. Other cardiotoxicities reported following ibrutinib use include arterial hypertension, ischemic events, and heart failure [44]. Arterial hypertension is a frequently reported adverse event following ibrutinib therapy, with a reported incidence of 78%, and can develop rapidly after initiation, making close monitoring essential in the early months of treatment [44-46]. Ibrutinib, along with other BTK inhibitors, may hinder the formation of blood clots on plaques that develop in arteries due to atherosclerosis. This effect is due to the interference of these inhibitors with platelet functions and their ability to aggregate, which can consequently lead to an elevated risk of bleeding [47]. Moreover, the concomitant use of ibrutinib and anticoagulants metabolized by the enzyme CYP3A4, such as apixaban, rivaroxaban, and dabigatran, can result in an increase in plasma concentrations and further augment the risk of bleeding [43].

Several studies have demonstrated elevated cardiac biomarkers after ibrutinib therapy [48,49]. Ciuculete et al. evaluated the ibrutinib cardiotoxicity in thirty-one patients after three months of therapy. Their findings showed that Tnt and NT-proBNP increased significantly (P = 0.019 and P = 0.03, respectively) after ibrutinib therapy compared to the control group [48]. Similarly, Mulder et al. demonstrated that ibrutinib has time-dependent effects on serum cardiac biomarkers in CLL [49]. Their findings showed that out of 86 changing biomarkers during ibrutinib treatment, 12 remained elevated throughout the treatment. Out of which six were related to AF or other cardiovascular diseases [49]. Similarly, a case of CLL patients who underwent ibrutinib therapy showed elevated troponin I levels (0.236 µg/L; reference: 0.00−0.07 µg/L) and NT-proBNP (2,798 pg/mL; reference: > 75 years old, < 450 pg/mL) [50].

Phosphoinositide 3-Kinases Inhibitors

The phosphoinositide 3-kinases (PI3K) pathway exerts critical control over multiple aspects of B cell physiology, governing key cellular processes including, proliferation, growth, migration, and apoptosis [51]. Currently, regulatory agencies such as the FDA and European Medicines Agency (EMA) have approved three inhibitors of the PI3K enzyme, namely idelalisib, copanlisib, and duvelisib, for treating indolent non-Hodgkin's lymphoma (NHL).

Idelalisib: Idelalisib is the first reversible and selective inhibitor of the delta isoform of PI3K, which plays a crucial role in the development and progression of NHL [52]. Although idelalisib has been associated with adverse effects such as pneumonia, hepatotoxicity, and skin rashes, cardiotoxicity is not that commonly reported [53,54]. However, some patients can experience atrial fibrillation and peripheral edema.

Copanlisib: Copanlisib is known to cause infusion-related hypertension (in 54.8% of cases, respectively), which typically develops within two hours of the first infusion cycle, causing an average increase in systolic blood pressure by 16.8 mmHg and resolves within 24 hours [55]. Although cardiotoxicity has been reported in a few studies in PI3K inhibitors, there is no available data to assess the utilization of serum cardiac biomarkers for cardiotoxicity for this targeted therapy. This leaves a significant gap in the monitoring and management of copanlisib-induced cardiotoxic effects. Further studies are warranted to explore this aspect and identify cardiac biomarkers related to PI3K inhibitors-induced cardiotoxicity. 

BCR/ABL (Tyrosine Kinase Fusion Protein) Inhibitors

The BCR/ABL fusion protein variants exhibit persistent activity in multiple signaling pathways, including PI3K and STAT [56].

Imatinib: Imatinib was the first drug to receive approval for the treatment of chronic myeloid leukemia (CML). Imatinib targets several proteins, including ABL, BCR/ABL, and c-KIT, in cancerous cells [57]. Imatinib targets specific kinases by binding to particular amino acid residues within the ATP binding site [58]. However, despite its intended specificity, imatinib can also affect unintended targets, leading to adverse effects such as cardiotoxicity. In patients with CML who receive imatinib treatment, chronic heart failure (CHF) can develop with New York Heart Association (NYHA) class 3-4 symptoms after an average of 7.2 ± 5.4 months of treatment [59]. Although rare, long-term imatinib treatment (six months or more) in older patients can result in CHF and left ventricular ejection fraction (LVEF) depression, with an incidence rate of 0.7-1.8% [60,61]. Herman et al. demonstrated a significant elevation in serum cardiac troponin I (cTnI) levels in rats treated with either 50 or 100 mg/kg of imatinib, in comparison to control groups [62]. The rise in cTnI levels was found to be positively correlated with the severity of tissue damage, with a dose-dependent increase observed in the treatment groups. Nevertheless, due to substantial inter-individual variability in cTnI values, none of the mean cTnI concentrations were significantly different from either the control or each other [62]. However, some studies have demonstrated the safety of imatinib. For example, Marcolino et al. conducted a pilot study involving twelve CML patients, with a mean follow-up period of 12.4 months, to investigate the potential impact of CML on cardiovascular health [63]. The study findings indicate that there were no statistically significant changes observed in the frequency of echocardiographic measurements, and BNP levels. Additionally, BNP levels remained relatively stable, with median levels of 8.3 pg/mL during baseline and 7.3 pg/mL during follow-up. Furthermore, troponin I measures were below the lower limit of detection, and strain measures were comparable to healthy controls [63]. Similar findings were shared by a single-center prospective study by of Francisco et al. in CML patients. They reported that there was no difference in cystatin-C and NT-proBNP before and after treatment [64]. However, a dose-dependent increase in cardiac biomarkers such as cTnI, cTnT, and FABP3 levels was noted with imatinib treatment by Herman et al. [65].

Nilotinib: Nilotinib is a potent inhibitor of BCR/ABL, but its therapeutic use is limited due to its cardiotoxicity, which causes QT prolongation and sudden cardiac death in a dose-dependent manner. These adverse effects are linked to the drug's off-target inhibition of the Kv11.1 cardiac potassium ion channel, which disrupts the QT interval [66]. Nilotinib also increases the risk of cardiovascular events, including acute coronary disease, myocardial infarction, and peripheral arterial occlusive disease, although the incidence varies [67]. Despite cardiotoxic effects, nilotinib is considered first-line therapy for the treatment of chronic myeloid leukemia. Furthermore, rosuvastatin can be an effective treatment for the cardiotoxicity of nilotinib [67]. Furthermore, nilotinib has pro-atherogenic effects on vascular tissue, leading to arterial stenosis and vasospasm. Additionally, it causes metabolic changes, such as elevated cholesterol and glucose levels, which contribute to an increased risk of cardiovascular disease [68]. Finally, nilotinib has a direct toxic effect on the heart by activating caspases and inducing apoptosis. The median time to a cardiovascular event is approximately 14.5 months, and patients may require multiple angioplasties and surgeries within a few months due to recurrent disease [69]. Chen et al., in a case report of a patient with CML, documented a significant increase in cardiac injury biomarkers after Nilotinib treatment. Specifically, the patient showed elevated levels of troponin I, myoglobin, and CK-MB. In addition, the patient's D-dimer and NT-proBNP levels were slightly elevated [70]. 

Bosutinib: Bosutinib is a second-generation tyrosine kinase inhibitor that specifically targets the SRC/ABL kinases and has been authorized for the treatment of CML [71]. Despite the relatively low incidence of cardiotoxicity associated with bosutinib at 6.8%, patients with refractory or relapsed CML who receive bosutinib as second- or above-line therapy have a higher risk of developing cardiac adverse events, especially cerebrovascular events, compared to those who receive bosutinib as first-line therapy (7.7% vs. 4.8%, respectively) [72]. In second- or above-line therapy, ECOG PS >, a history of vascular disorders, hypercholesterolemia, and age ≥65 years were significant risk factors for cardiovascular adverse events [72]. The most commonly observed cardiotoxic manifestations associated with bosutinib include angina pectoris, coronary artery disease (CAD), and peripheral artery disease (PAD), with an incidence of 1.2%, 1.2%, and 2%, respectively [72]. Hypertension is another potential adverse effect associated with bosutinib, occurring in 7.8% of cases, especially in those with a history of hypertension. Nevertheless, the incidence of hypertension with bosutinib is similar to that observed with other tyrosine kinase inhibitors, such as imatinib, in patients with CML [72]. Bras et al. in their study showed that serum NT-proBNP was increased 2 fold in patients treated with bosutinib at 24 h after three sequences [73]. Similarly, Demeter et al. showed an increased level of cardiac biomarkers in CML patients treated with 500 mg bosutinib/day [74].

Ponatinib: Ponatinib is a third-generation tyrosine kinase inhibitor that targets multiple kinases, including SRC/ABL, FGFR1, and VEGFR2 [75]. However, it is associated with cardiotoxicity, including CHF, arrhythmias, and hypertension [76-79]. In addition, ponatinib can enhance platelet activation and aggregation, promote surface adhesion receptor expression, and have pro-atherogenic properties, leading to a high incidence of coronary artery disease (CAD), peripheral artery disease (PAD), and cerebrovascular events [79]. Madonna et al. investigated ponatinib-related cardiotoxicity and showed a fourfold increase in cardiac biomarker BMI1 in treated female rats [80]. BMI1 is a protein marker that promotes cell survival and has a crucial function in responding to DNA damage, as well as in regulating mitochondrial function and maintaining a balance of reactive oxygen species (ROS). Increased expression of BMI1 in cardiac tissue is recognized as an indicator of the heart's ability to repair itself following injury [81-83].

Proteasome Inhibitors

Proteasome inhibitors that target the activity of proteasomes can impede the progression of cancer by hindering the activity of both the constitutive and immune proteasome [84].

Bortezomib: Bortezomib, the first proteasome inhibitor to be developed, shows activity against both forms of proteasome by targeting specific subunits (β5, β1, and β5i) [85]. However, the inhibition of proteasomes can lead to cardiomyocyte dysfunction and heart failure, as proteasomes play a crucial role in maintaining protein homeostasis in cardiac tissues [86]. In rats, bortezomib has been shown to induce left ventricular contractile dysfunction with mitochondrial modifications, as well as reductions in ATP synthesis and cardiomyocyte contractile functions [87]. Clinical trials have revealed that the incidence of bortezomib-associated cardiotoxicity ranges from 0% to 17.9%, with the highest incidence observed in elderly patients with mantle cell lymphoma (MCL) and in patients with multiple myeloma (MM) who received bortezomib as monotherapy [88]. The most common form of cardiotoxicity observed is CHF, with a hospitalization rate of 5.76/100 patient-years, particularly in patients aged over 70 years, and after a median of 3.2 months following the initiation of bortezomib treatment [89]. Diwadkar et al. described a clinical case of a 66-year-old male patient with multiple myeloma who was treated with bortezomib and had consistently elevated levels of cardiac biomarkers, despite being asymptomatic [90]. Following his eighth cycle of treatment with bortezomib, the patient developed a complete heart block and evidence of myocardial scarring, necessitating permanent pacemaker placement. The patient's cardiac biomarkers, including troponin I, CK-MB, and creatine phosphokinase, were found to be elevated, with a troponin I level of 2.49 ng/mL, CK-MB of 20.2, and creatine phosphokinase of 549. Additionally, the patient's BNP level was also elevated, measuring 333 pg/mL [90]. Similarly, Alali and Baljevic reported a case of peri myocarditis induced by bortezomib in a patient with multiple myeloma. The patient's laboratory results revealed elevated N-terminal pro-B-type natriuretic peptide levels and normal troponin I levels [91].

Carfilzomib: Carfilzomib, a second-generation proteasome inhibitor, has been found to exhibit cardiotoxic properties akin to bortezomib. Notably, the incidence of carfilzomib-induced cardiac events is greater in comparison to bortezomib-treated patients, with a rate of 27% and 16%, respectively [92,93]. Carfilzomib-induced cardiotoxicity is caused by the inhibition of AMPKα-mediated autophagy and PI3K/Akt/eNOS axis [94]. Rosenthal et al. showed that NT-proBNP were frequently elevated post-treatment with carfilzomib, often without corresponding cardiopulmonary symptoms [95]. In a study conducted by Atrash et al., they investigated the cardiac complications in relapsed and refractory multiple myeloma patients who were treated with carfilzomib. Of the 130 patients involved in the study, 69 of them had their baseline BNP levels measured, as well as, measurements taken during the first cycle of carfilzomib treatment. The results showed a significant median increase of 407 pg/mL in BNP levels from baseline (P<0.001) [96]. In a study conducted by Cornell et al., 95 patients were enrolled and followed up for a median of 25 months. Among them, 65 received carfilzomib and 30 received bortezomib. The study found that 64 cardiovascular adverse events (CVAEs) occurred during the follow-up period, with 55% of them being grade 3 or greater in severity. The incidence of CVAEs was significantly higher in patients treated with carfilzomib-based therapy (51%) compared to those treated with bortezomib (17%) (P = .002). Furthermore, the study identified that patients with elevated baseline levels of BNP higher than 100 pg/mL or N-terminal proBNP level higher than 125 pg/mL had a significantly increased risk of developing CVAEs (odds ratio, 10.8; P < .001) [97].

Immune Checkpoint Inhibitors

Immune checkpoint inhibitors (ICI) are a class of antibodies that activate the immune system by targeting immune checkpoints. Although these agents have demonstrated remarkable clinical benefits in various cancers, they can also lead to adverse cardiac events, including myocarditis, vasculitis, pericarditis, cardiac conduction disease, non-inflammatory LVD, and HF. The mechanisms underlying ICI-related cardiotoxicity are still unclear; however, immune-mediated myocarditis is considered a result of an exaggerated adaptive immune response against shared epitopes in the myocardium and tumor cells [98,99]. In the case of myocarditis associated with ICI therapy, an increase in troponin levels may occur, even in subclinical forms of the disease. Furthermore, a multi-center study by Mahmood et al. reported that there was a four-fold increased risk of major adverse cardiac events with troponin T of ≥1.5 ng/ml (P = 0.003) in individuals treated with ICIs [100]. Some studies have reported incidences of myocarditis ranging from 0.3% to greater than 1% in patients receiving ICI therapy, especially in combination with other agents [101,102]. Early onset of symptoms, frequent mortality, and a dramatic increase in cases reported in 2017 are common in these cases [102]. Diagnosis of cardiotoxicity is usually made using a combination of biomarkers, cardiac imaging, and biopsy. A specific definition of ICI-related myocarditis has been recently published, including elevated cTn as a key variable for diagnosis and trigger for more advanced investigations [103]. Due to the fulminant nature and higher frequency of ICI-associated myocarditis in patients receiving combination therapy, various screening protocols have been proposed, including the use of cardiac biomarkers and ECG. Mahmood et al. showed that the patients with myocarditis had an elevation in troponin (94%) and had an abnormal ECG (89%) [100]. Similarly, Escudier et al. showed that BNP or NT-proBNP concentrations were elevated in 70-100% of patients [101]. Additionally, patients suspected of having ICI-associated myocarditis should be assessed for myositis, including checking for creatine kinase [104].

Chimeric Antigen Receptor T-cell Therapy

Chimeric antigen receptor T-cell (CAR-T) therapy is a new approach for treating relapsed or refractory hematologic malignancies such as leukemia and lymphoma. Recent reports have indicated that cardiotoxicity is one of the side effects associated with this therapy. The precise mechanism underlying this toxicity remains unclear, but it is believed to involve both direct and indirect toxicity. CAR-T cells may recognize and react with proteins that are similar to the target antigen in normal tissues, leading to direct toxicity. Indirect toxicity may also result from the cytokine release syndrome mechanism [105]. Cytokine release syndrome (CRS) is the indirect cardiotoxicity of CAR-T cell therapy caused by a release of cytokines by the infused CAR-T cells, resulting in systemic inflammation [105]. These effects can cause a decrease in left ventricular systolic function, hypotension, arrhythmia, and prolongation of the QTc interval. A recent study by Alvi et al. reported that out of 53 patients who received CAR-T therapy, 29 (54%) had elevated levels of troponin [106]. Therefore, monitoring troponin levels is recommended to identify patients who may be at risk of cardiotoxicity during CAR-T therapy [2]. Table 1 summarizes targeted therapies and their cardiotoxic effects and serum cardiac biomarkers.

**Table 1:** Summary of cardiotoxicities associated with different targeted cancer therapies and their associated serum cardiac biomarkers

Targeted therapy	Agent	Indications	Cardiotoxicity	Serum Cardiac Biomarkers	References
Bruton’s Tyrosine Kinase Inhibitors	Ibrutinib	Mantle cell lymphoma (MCL), Chronic lymphocytic leukemia (CLL), Waldenström’s macroglobulinemia	Atrial fibrillation, ventricular arrhythmias, hypertension, bleeding	Elevated Tnt and NT-proBNP	[48], [50]
Phosphoinositide 3-kinases (PI3K) inhibitors	Copanlisib	Relapsed follicular lymphoma (FL)	Hypertension, decreased left ventricular ejection fraction, QT interval prolongation	Not specified	[55]
BCR/ABL Inhibitors	Imatinib	Chronic Myeloid Leukemia	Mild to moderate, reversible QT prolongation, rare cases of heart failure and sudden death	No significant changes	[63], [64]
				Elevated serum cardiac troponin I (cTnI)	[62], [65]
	Nilotinib	Chronic Myeloid Leukemia	Moderate to severe, reversible QT prolongation, rare cases of heart failure and sudden death	Elevated troponin I and NT-proBNP	[70]
	Bosutinib	Chronic Myeloid Leukemia	Mild to moderate, reversible QT prolongation	Elevated NT-proBNP	[74]
	Ponatinib	Chronic Myeloid Leukemia	Moderate to severe, arterial thrombotic events, hypertension, heart failure, QT prolongation	Elevated BMI1	[80]
Proteasome Inhibitors	Bortezomib	Multiple Myeloma	Left ventricular contractile dysfunction with mitochondrial modifications	Elevated troponin I, CK-MB, and creatine phosphokinase	[90], [91]
	Carfilzomib	Multiple Myeloma	Hypertension, heart failure, arrhythmias	NT-proBNP, BNP (elevated)	[95], [96]
Immune Check point inhibitors	Ipilimumab, Nivolumab, Pembrolizumab, Atezolizumab, Durvalumab, Avelumab, Tremelimumab	Advanced melanoma, NSCLC, RCC, Hodgkin lymphoma, HNSCC, urothelial carcinoma	Myocarditis, Pericarditis, heart failure, arrhythmias	Elevated BNP, NT-proBNP	[101]
Chimeric antigen receptor T-cell therapy	Brexucabtagene autoleucel, Idecabtagene vicleucel, Ciltacabtagene autoleucel, Abecma, Breyanzi.	Mantle cell lymphoma, Acute lymphoblastic leukemia, Multiple myeloma	Left ventricular systolic function, hypotension, arrhythmia, and prolongation of the QTc interval.	Elevated levels of troponin	[106]

## Conclusions

Targeted therapies have revolutionized cancer treatment, but their use has been associated with cardiac toxicity, leading to morbidity and mortality. The detection and monitoring of cardiac biomarkers can play a significant role in the early detection of cardiotoxicity and guide the modification of treatment strategies to mitigate adverse cardiovascular outcomes. Bruton’s tyrosine kinase inhibitors, phosphoinositide 3-kinases inhibitors, BCR/ABL inhibitors, proteasome inhibitors, immune checkpoint inhibitors, and chimeric antigen receptor T-cell therapy are the commonly used targeted therapies with a potential for cardiac toxicity. The identification of specific serum biomarkers associated with the different targeted therapies can help in risk stratification and management of cardiovascular complications. There is a need for larger prospective studies to validate the clinical utility of the emerging serum biomarkers in the cardiological risk stratification of patients with hematological malignancies. Identifying specific biomarkers associated with each targeted therapy is crucial in the early detection and management of cardiotoxicity. Furthermore, the integration of cardiac biomarkers in the routine care of patients with lymphoproliferative disorders treated with targeted therapies can help in reducing cardiovascular morbidity and mortality.
